# Stapler port position determination using 3-dimensional virtual simulation software in robot-assisted thoracic surgery

**DOI:** 10.1186/s12893-023-02112-5

**Published:** 2023-07-21

**Authors:** Satoshi Hayashi, Masaya Kawada, Yuki Mori

**Affiliations:** 1 Department of Respiratory and Breast Surgery, Tonan Hospital, Kita 4 Nishi 7, Chuo-Ku, Sapporo, Hokkaido 060-0004 Japan; 2 Radiologic Technology Department, Tonan Hospital, Kita 4 Nishi 7, Chuo-Ku, Sapporo, Hokkaido 060-0004 Japan

**Keywords:** Stapler port, 3-dimensional virtual simulation software, Robot-assisted thoracic surgery, SureForm 45 Curved-Tip stapler

## Abstract

**Background:**

In robot-assisted thoracic surgery (RATS) lobectomy using a robotic stapler, stapling is difficult when the stapler port place is close to the resection target vessel. We examined whether three-dimensional computed tomography (3D-CT) software enables stapler port place determination for stapling.

**Methods:**

Seventy-three patients who underwent RATS lobectomy were enrolled. The SureForm 45 Curved-Tip stapler (136 mm from the remote center to the anvil tip) was used. The virtual distance between the resection target vessel and stapler port place (VD) was preoperatively measured with 3D-CT software. The stapler port place was the most cranial intercostal space with a VD ≥ 136 mm. The actual distance between the resection target vessel and anvil tip (AD) was measured intraoperatively. We examined the associations of the difficulty in stapling with VD, AD, chest wall damage, and clinical features.

**Results:**

Stapling was easier with a larger anteroposterior thoracic diameter and AD. The cut-off VD and AD for smooth stapling were 142 mm and 6 mm. Chest wall damage was frequently observed at the caudal and dorsal side ports.

**Conclusions:**

As the stapler port place is located more caudally, stapling becomes easier. However, chest wall damage increases. If the stapler port place is positioned at a site ensuring VD ≥ 142 mm by 3D-CT software, smooth stapling may be possible with a decreased incidence of chest wall damage.

## Introduction

**Fig. 1:**
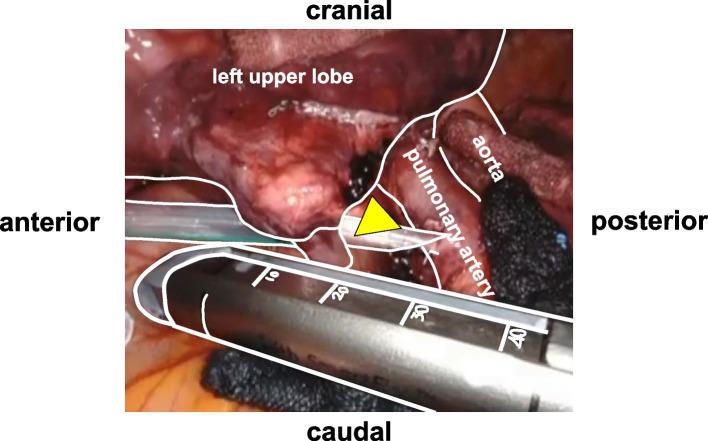
The anvil tip was located on the cranial side from the pulmonary vein at the time of intrathoracic insertion of the stapler. Arrowhead: Left lingular vein (V^4^ + V.^5^)

**Fig. 2:**
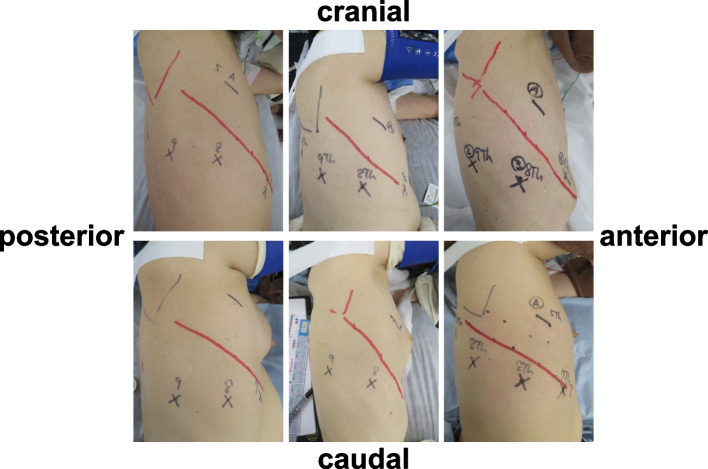
Intercostal running variation (right side). The angle and dimensions of the ribs vary, and it can be assumed that the distance to the hilar is different even between the same intercostal muscles. Red line: Seventh rib

In robot-assisted thoracic surgery (RATS), the stapler port place selection is important. Robotic staplers have a wide range of motion and the advantage of allowing surgeons to perform stapling without assistance. However, when the stapler port place is close to the resection target vessel, stapling is difficult (Fig. 1). Compared to Europeans and Americans, Asians have a smaller body constitution, and variable thorax shapes (Fig. 2). Thus when the stapler port place is determined using a method based on the intercostal space, such as the method recommended by Intuitive Surgical [1], the distance between the stapler port place and resection target vessel may be insufficient in some Asian patients. With a focus on this distance, we devised a method to determine the stapler port place by measuring the distance between the resection target vessel and stapler port place (virtual distance between the resection target vessel and stapler port: VD) with three-dimensional computed tomography (3D-CT) software (SYNAPSE VINCENT medical imaging system, Fujifilm Medical, Tokyo: VINCENT). VINCENT enables surgeons to analyze 3D-CT data easily and quickly for various purposes, such as determination of the distance to, and the courses of, arteries, veins, and bronchi, measurement of the volume of resected lung, and setting of the resection surfaces of the lung. This study aimed to reveal whether our distance-based method allows for objective selection of stapler port place for smooth stapling and whether the cut-off VD necessary for smooth stapling can be determined.

## Methods

### Patients, preoperative stapler port place determination and surgical procedures

Between May 2021 and April 2023, seventy-three consecutive patients who underwent lobectomy at Tonan Hospital using the da Vinci Xi (Intuitive Surgical, Sunnyvale, CA) were included in this study. Using preoperative 3D-CT data and the lung resection analysis application of VINCENT, we selected multiple intercostal spaces that were candidates for the stapler port place, and then three-dimensionally measured the linear distance to the most caudal resection target vessel. For this method, the most caudal vessel was defined as the superior lobar vein in the upper lobe, the middle lobar vein in the middle lobe, and the inferior lobar vein in the lower lobe.

The patient was positioned in lateral decubitus and single-lung anesthesia induced via a double lumen tube. The operating table was flexed to widen the intercostal space. The da Vinci Xi was positioned behind the patient. We performed surgery based on the completely portal four-arm robotic technique [1]. In all patients, the AirSeal® (ConMed, Utica, NY) was simultaneously used for CO_2_ insufflation at 6 mmHg.

**Fig. 3:**
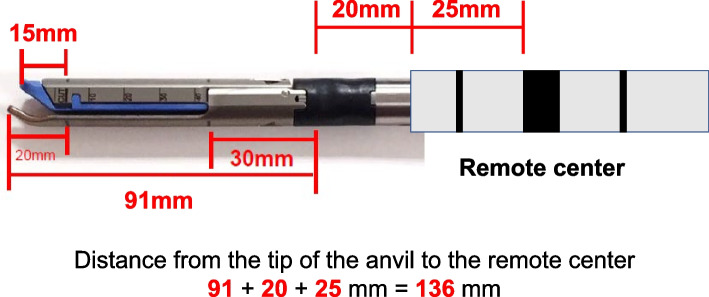
The SureForm 45 Curved-Tip specification

**Fig. 4:**
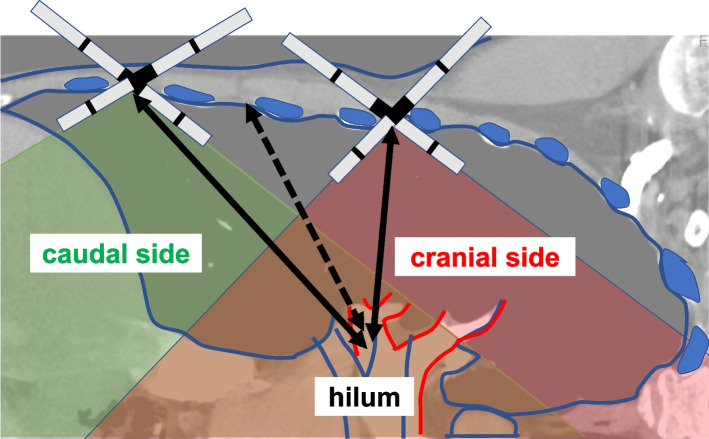
Differences in operability in the chest cavity due to port position (right). Two-headed arrow: Distance between port and hilum. Two-headed arrow (dotted): Ideal distance between the port and hilum. Green area: Range of motion of the instrument at the caudal side port. Red area: Range of motion of the instrument at the cranial side port

Only the SureForm 45 Curved-Tip (Intuitive Surgical, Sunnyvale, CA) was used as the stapler. As the actual measurement of the length from the remote center to the anvil tip of the SureForm 45 Curved-Tip was 136 mm (Fig. 3), (1) the first criterion for selection of stapler port place was an intercostal space with a VD of 136 mm or more. When the acquisition of a VD of 136 mm resulted in the placement of the stapler port place in the abdominal cavity, the stapler port place was set in the most caudal intercostal space in the thoracic cavity. Although stapling is easier with a more caudally placed stapler port place, the use of other instruments may limit the operable range in the thoracic cavity and cause chest wall damage or interference with bones (Fig. 4). Thus, (2) the second criterion was a stapler port place located as cranially as possible. While we set the standard stapler port place and other da Vinci ports at the eighth intercostal space in accordance with the method recommended by Intuitive Surgical [1], the standard stapler port place determined by this intercostal method was changed as needed to meet criteria (1) and (2). The superior lobar vein and the middle lobar vein were stapled from the second port from the posterior, and the inferior lobar vein was stapled from the most anterior port. An assistant port was placed in the fourth or fifth intercostal space near the hilum in preparation for sudden bleeding.

### Evaluation of the difficulty in stapling and operative data

The difficulty in stapling was determined after scoring as follows. When the resection target vessel can be easily cut without flexing the staple a score of one point was given. Two points were appropriate for cases that the resection target vessel can be cut by flexing the stapler. Three points were given for cases that the anvil tip is close to or over the resection target vessel even if the stapler is flexed. All 73 patients were scored by four thoracic surgeons, and cases with a total score of 4–6 were classified as easy, 7–9 as typical, and 10–12 as impossible/difficult. Stapling classified as the typical or easy level was considered to indicate the possibility of smooth stapling. Furthermore, unexpected bleeding requiring cauterization and pleural damage around the da Vinci port site was counted as chest wall damage events.

**Fig. 5:**
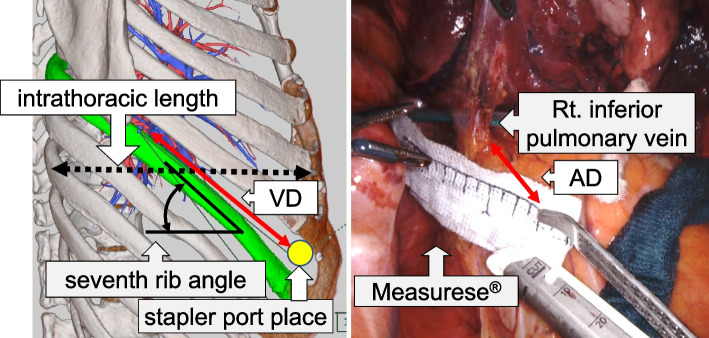
Clinical indicators. VD: Virtual distance between the resection target vessel and stapler port place. AD: Actual distance between the resection target vessel and the anvil tip

**Fig. 6:**
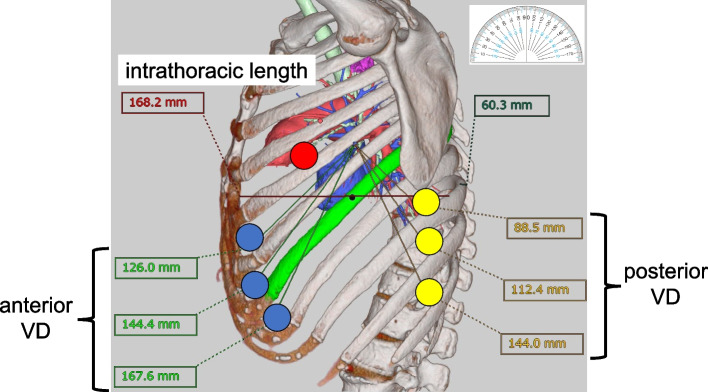
Our distance-based method in the left upper lobectomy. The distance between multiple intercostal spaces and the resection target vessel candidates for stapler port place is measured by VINCENT. In this case, the sixth intercostal space (VD = 144.4 mm) was selected as the anterior stapler port place, and the tenth intercostal space (VD = 144.0 mm) was selected as the posterior stapler port place. VD: Virtual distance between the resection target vessel and stapler port place. Red circle: Assistant port place. Blue circle: Candidates for the anterior stapler port place. Yellow circle: Candidates for the posterior stapler port place. Green: Seventh rib

Age, sex, height, weight, body mass index, length from the backside of the sternum to the most posterior aspect of the thoracic cavity on CT slices depicting the seventh rib at the most lateral part (intrathoracic length), seventh rib angle, VD, and the intraoperatively measured distance between the resection target vessel and anvil tip (actual distance between the resection target vessel and the anvil tip [AD] that was measured with Measurese® [Hakuzo Medical, Osaka, Japan] while the stapler was kept straight) were examined to determine the associations with the stapling difficulty. Figure 5 shows the measurement methods for each item, and Fig. 6 shows our method in the left upper lobectomy.

### Statistical analysis

Numerical data (age, height, weight, body mass index, intrathoracic length, seventh rib angle, VD, and AD) were treated as continuous variables and other categorical variables. Chi-square or Fisher’s exact test was used to determine significant differences in categorical variables, and the Mann–Whitney U test was used to compare the distributions of continuous variables for univariable analysis. A significant difference was predetermined to be a *P*-value less than or equal to 0.05. The post hoc Tukey–Kramer honestly significant difference test was performed when differences were found. Receiver operating characteristic (ROC) curve analysis was performed to determine the optimal cut–off values for VD and AD. Statistical analyses were performed using JMP® 10 (SAS Institute Inc., Cary, NC, USA).

## Results

### Patients characteristics

This study included 36 men and 37 women with a mean age of 70 years, height of 160 cm, weight of 56 kg, body mass index of 21.7, intrathoracic length of 164.3 mm, seventh rib angle of 39°, VD of 145.0 mm, and AD of 11.7 mm. In all 73 patients, stapling of the resection target vessel was completed with only the SureForm 45 Curved-Tip. According to our method, the standard stapler port place was changed to be placed at the caudal side in 47 patients. There were no patients in whom the standard stapler port place was changed to be placed at the cranial side. No significant differences in clinical features or resected pulmonary lobes were observed between patients with and without changes in the standard stapler port place (data not shown).

### Evaluation of the difficulty in stapling

**Fig. 7:**
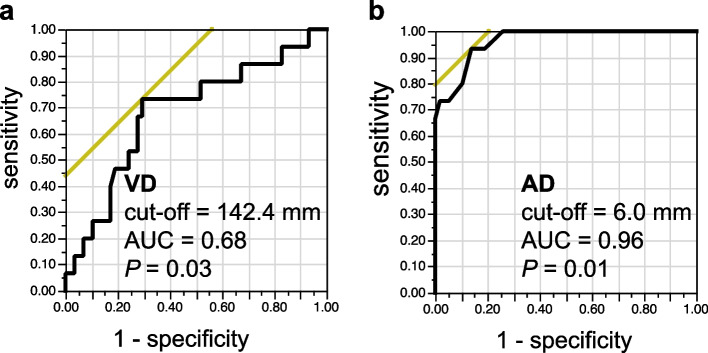
Receiver operating characteristic curve analysis to determine the optimal cut-off value of the VD (a), and AD (b) for predicting the stapling difficulty. VD: Virtual distance between the resection target vessel and stapler port place. AD: Actual distance between the resection target vessel and the anvil tip. AUC: Area under the curve

Table 1 shows the difficulty in stapling. According to the previous scoring classification, there were 16 Impossible/Difficult cases, 25 Typical cases, and 32 Easy cases. Stapling was easier with larger intrathoracic length (Impossible/Difficult; Typical; Easy = 161; 159; 170 mm, *P* = 0.005. Significant difference between Impossible/Difficult/Typical and Easy.) and AD (Impossible/Difficult; Typical; Easy = -3.5; 9.4; 21.0 mm, *P* < 0.001. Significant difference between all groups.). Although no significant difference was observed in VD, stapling was easier with a larger VD (Impossible/Difficult; Typical; Easy = 140.6; 144.4; 147.8 mm, *P* = 0.11). Since the value resulting from the subtraction of the length of the SureForm 45 Curved-Tip (136 mm) from VD (VD-136) appears to be identical to AD, the means of both values were examined. The mean VD-136 was 9.0 mm, and the mean AD was 11.7 mm. A significant difference was not observed between them (*P* = 0.14). VD was weakly correlated with height (*r* = 0.45) and weight (r = 0.36), and AD was weakly correlated with intrathoracic length (*r* = 0.31). A weak correlation was also observed between VD and AD (*r* = 0.35). The cut-off values for VD and AD necessary for smooth stapling were 142.4 mm (Area under the curve [AUC]: 0.68. *P* = 0.03) and 6.0 mm (AUC: 0.96. *P* = 0.01), respectively (Fig. 7). Chest wall damage was observed at 49 ports in 44 of the 73 patients, and its incidence was higher at the caudal and posterior side ports (Table 2).

**Table 1:** Relationship between clinical features and stapling difficulty

Variables		Stapling difficulty		*P*-value
	Impossible/Difficult (*n* = 16)	Typical (*n* = 25)	Easy (*n* = 32)	
Age, y	70 ± 8 [66, 75]	71 ± 8 [67, 74]	69 ± 10 [65, 72]	0.63
Male	6 (38)	11 (44)	19 (59)	0.29
Height, cm	159 ± 9 [155, 163]	159 ± 7 [155, 162]	161 ± 9 [158, 164]	0.45
Weight, kg	53 ± 10 [48, 58]	54 ± 10 [50, 58]	58 ± 10 [55, 62]	0.17
Body mass index, kg/m^2^	21 ± 3 [19, 23]	21 ± 4 [20, 23]	22 ± 3 [21, 24]	0.41
Intrathoracic length, mm	161 ± 16 [154, 167]	159 ± 15 [154, 164]	170 ± 9 [166, 175]	0.005*
Seventh rib angle, degree	39 ± 7 [35, 43]	40 ± 9 [37, 43]	38 ± 7 [35, 40]	0.60
VD, mm	140.6 ± 12.2 [135.1, 146.1]	144.4 ± 10.4 [140.0, 148.8]	147.8 ± 11.2 [143.8, 151.7]	0.11
VD-136, mm	4.6 ± 12.2 [-0.9, 10.2]	8.4 ± 10.4 [3.9, 12.8]	11.8 ± 11.2 [7.8, 15.7]	0.11
AD, mm	-3.5 ± 10.5 [-7.9, 0.9]	9.4 ± 4.6 [5.9, 12.9]	21.0 ± 10.3 [17.9, 24.1]	< 0.001**

Values are mean ± standard deviation [95% Confidence interval], n (%)

^*^Significant difference between Impossible/Difficult/Typical and Easy

^**^Significant difference between all groups

Intrathoracic length: length from the sternum to the inside of the rib with the seventh rib arranged in the outermost computed tomography image

VD: Virtual distance between the resection target vessel and stapler port

AD: Actual distance between the resection target vessel and the anvil tip

**Table 2:** Relationship between port sites and chest wall damage

		← anterior arm			posterior arm →
		**arm 1**	**arm 2**	**arm 3**	**arm 4**
intercostal space	**6**^**th**^	1 / 30 3%	0 / 0	0 / 0	0 / 0
	**7**^**th**^	3 / 43 7%	0 / 11	0 / 0	0 / 0
	**8**^**th**^	0 / 0	4 / 60 7%	1 / 2 50%	6 / 10 60%
	**9**^**th**^	0 / 0	0 / 2	10 / 51 20%	20 / 60 33%
	**10**^**th**^	0 / 0	0 / 0	3 / 20 15%	1 / 3 33%

Values are event (n) / port (n), %

## Discussion

Stapling is the most dangerous step of lobectomy. In early versions, before the da Vinci Xi system was introduced, stapling had to be performed by an assistant, meaning that an experienced thoracic surgeon was needed at the bedside. Moreover, there are also associated safety issues because stapling is performed between the da Vinci arms [2, 3]. One advantage of RATS is the possibility of solo surgery. Although the da Vinci stapler is undeniably useful in this regard, its features need to be better understood.

As shown in Fig. 1, it is difficult to pull the da Vinci stapler out of the thoracic cavity from the intrathoracic position where the stapler is initially guided. Although there are numerous methods, the standard method is to place a da Vinci port in an intercostal space, which is used as a rough indication [1, 4, 5]. However, in Asians, who have a small body constitution and whose thorax varies in shape (Fig. 2), even the selection of the same intercostal space causes variations in the distance between the hilum, where the resection target vessels are concentrated, and the port. Thus, although the intercostal space was used as a principal site for the stapler port place, we decided to set the stapler port place at a site where a necessary and sufficient distance for stapling could be guaranteed based on the results of the preoperative distance-based method. After this method was performed, the stapler port place was changed to be placed at a more caudal side than the standard stapler port place in the majority of the patients. If the stapler port place was determined by the intercostal space method without performing our method, the resection target vessel may be close to the stapler port place, as shown in Fig. 1, and stapling may be difficult in some patients.

Pearlstein et al. [2] recommend that the stapler port place should be placed as caudally as possible when the da Vinci stapler is used. As the setting of the stapler port placed at the caudal side increases AD, stapling may be easier. Conversely, in RATS, the stapler port is used for not only a stapler but also other instruments. When this port is placed at the caudal side, for example, dissection of the caudal aspect of the hilar region can be performed without any problem, whereas dissection of the cranial aspect, lymph node dissection in the upper mediastinum, and other procedures performed by crossing over the hilum are difficult to perform even with da Vinci instruments with a wide range of motion. Moreover, dissection of the adhered diaphragm is difficult to perform from the caudal side port, and many thoracic surgeons have experienced chest wall damage and interference with bones that are caused by directing instruments toward the cranial side. In fact, chest wall damage occurred frequently at the caudal and posterior side ports in this study. Based on the above, we consider that performing this distance-based method to place the stapler port at possibly the most cranial site with a sufficient distance to resection target vessel is important when performing smooth stapling without complications. Based on the ROC curve analysis of the cut-off value, a VD of 142 mm may allow easy stapling. The reason that AD (mean 11.7 mm) was slightly larger than VD-136 (mean 9.0 mm) was considered to be the effect of CO_2_ insufflation at 6 mmHg by AirSeal® and the gravitational effect of the lateral position. In addition, intrathoracic length had the possibility of predicting stapling difficulty other than VD and AD, but it was not possible to separate the Impossible/Difficult group from the Typical group. Furthermore, general indicators such as sex, height, and weight provide insufficient information on the stapler port place determination and stapling difficulty prediction.

In the situation shown in Fig. 1, ligation is the easiest procedure. However, stapling is necessary when resection target vessels are large. The possible coping techniques include 1) the use of a 30-mm da Vinci stapler (EndoWrist), 2) the addition of a port at a site where stapling is possible, 3) the use of a stapler by an assistant at the bedside, and 4) stapling with the port slightly pulled out of the thoracic cavity. Technique 1 is costly and time-consuming; furthermore, the EndoWrist has a narrower range of motion than the SureForm. Techniques 2 and 3 increase the number of wounds and require a skilled thoracic surgeon to be present at the bedside. Since technique 4 deviates from the location of the remote center, this technique is associated with a safety issue and should be avoided. Performing safe surgery should be the most important point, and the cost or the number of wounds is of lesser concern. Performing our method before surgery can avoid situations that would otherwise require techniques 1 to 4. With the 3D-CT software being widely used, this method has the advantages of being objective and simple (can be performed in 5 min).

The limitations of this study are the small sample size of Impossible/Difficult cases, which resulted from a single-center study. The surgical procedure was the subject, and it was difficult to objectively evaluate the difficulty in stapling and decreased operability at the caudal side port. RATS has been widely introduced in Japan relatively recently, and the small sample size is unavoidable. A study with many cases in multiple institutions is necessary to confirm our results. In addition, Japan is relatively racially uniform compared to other countries, it is unclear how useful our distance-based method is in other populations. Although it is difficult to say that the 3D-CT software is widely used internationally, we hope to conduct international research that can confirm the usefulness of our method in the future. Our method alone may not be sufficient in determining the appropriate stapler port place. However, we consider our method to be useful, as lobectomy was completed only with the SureForm 45 Curved-Tip in all 73 patients. We will continue to accumulate cases and to further evaluate this method.

## Conclusion

As the stapler port place is located more caudally, stapling becomes easier. However, chest wall damage increases. If the stapler port place is positioned at a site ensuring VD ≥ 142 mm by 3D-CT software, smooth stapling may be possible with a decreased incidence of chest wall damage. An appropriate stapler port place can be objectively selected by performing our distance-based method.

## Data Availability

The datasets of the current study are available from the corresponding author upon reasonable request.
